# Total and Free Hydrogen Cyanide Content and Profile of Bioactive Amines in Commercial *Tucupi*, a Traditionally Derived Cassava Product Widely Consumed in Northern Brazil

**DOI:** 10.3390/foods12234333

**Published:** 2023-12-01

**Authors:** Brenda de Nazaré do Carmo Brito, Mayara Galvão Martins, Renan Campos Chisté, Alessandra Santos Lopes, Maria Beatriz Abreu Gloria, Rosinelson da Silva Pena

**Affiliations:** 1 Graduate Program of Food Science and Technology (PPGCTA), Institute of Technology (ITEC), Federal University of Pará (UFPA), Belém 66075-110, PA, Brazil; brendabrito07@gmail.com (B.d.N.d.C.B.); alessalopes@ufpa.br (A.S.L.); 2 Innovation, Development and Adaptation of Sustainable Technologies Research Group (GPIDATS), Mamirauá Institute for Sustainable Development (IDSM), Tefé 69553-225, AM, Brazil; mayara.martins@mamiraua.org.br; 3 Faculty of Food Engineering (FEA), Institute of Technology (ITEC), Federal University of Pará (UFPA), Belém 66075-110, PA, Brazil; 4 Laboratory of Food Biochemistry-LBqA & LCQ, Faculty of Pharmacy, Federal University of Minas Gerais (UFMG), Belo Horizonte 31270-901, MG, Brazil; mbeatriz.gloria@gmail.com

**Keywords:** *manipueira*, cassava products, hydrogen cyanide, biogenic amines, polyamines

## Abstract

*Tucupi* is a broth derived from cassava roots which is produced after the spontaneous fermentation of *manipueira* (the liquid portion obtained by pressing cassava roots), followed by cooking. This product is widely consumed along with traditional dishes in the Brazilian Amazonia and is already used in different places worldwide. In this study, *tucupi* obtained from the markets of Belém (Pará, Brazil) and produced using agroindustrial (11 samples) and non-agroindustrial (11 samples) units were investigated to determine their physicochemical characteristics, total and free HCN contents, and free bioactive amine profiles. Most of the samples showed significant variations (*p* ≤ 0.05) in pH (2.82–4.67), total acidity (0.14–1.36 g lactic acid/100 mL), reducing sugars (up to 2.33 g/100 mL), and total sugars (up to 4.35 g/100 mL). Regarding the amines, four biogenic amines (0.5–4.2 mg/L tyramine, 1.0–23.1 mg/L putrescine, 0.5–66.8 mg/L histamine, and 0.6–2.9 mg/L tryptamine) and one polyamine (0.4–1.7 mg/L spermidine) were identified in the *tucupi* samples. Even in the *tucupi* produced using the agroindustrial units, which had quality seals provided by the local regulatory agency, high levels of biogenic amines (4.4–78.2 mg/L) were observed, as well as high dosages of total (8.87–114.66 mg/L) and free (0.80–38.38 mg/L) HCN. These facts highlight the need for better knowledge regarding the product manufacturing process to establish standardization and high-quality conditions for *tucupi* processing since high contents of biogenic amines and HCN are commonly associated with adverse health effects.

## 1. Introduction

Cassava (*Manihot esculenta* Crantz) is one of the main food crops in the world; 314 million tons are produced per year, and it is widely cultivated by family farmers in Brazil [1,2]. Cassava roots are traditionally used in Northern Brazil to prepare several foodstuffs, such as flour and starch, its main products.

In the production of cassava flour, the roots are crushed and pressed, and the liquid portion, which is called *manipueira*, and which contains the starch, is obtained. The starch is recovered via decantation, and the liquid supernatant can be subjected to spontaneous fermentation for up to 24 h, after which it can be cooked to obtain *tucupi*, another cassava product widely used in Amazon cuisine [3,4].

*Tucupi* is a broth commonly used in traditional Brazilian Amazonian cuisine which is derived from the spontaneous fermentation of *manipueira*, the liquid portion obtained by pressing cassava roots [5,6]. The fermentation process not only influences the flavor and texture of the *tucupi*, but it is also responsible for its preservation. Organic acids produced during fermentation act as natural preservatives, increasing the shelf life of *tucupi* and contributing to its food safety [7,8]. During the fermentation of *tucupi*, microorganisms found in cassava and *manipueira*, such as lactic acid bacteria and yeasts [9,10], are involved in the transformation of *manipueira* into *tucupi*. These microorganisms play a fundamental role in the production of compounds that give *tucupi* unique sensory characteristics, including its acidic flavor, its aroma, and its characteristic yellowish color [4,11]. This delicacy is an essential component in many traditional dishes from Northern Brazil and contributes to the gastronomic identity of the Brazilian Amazon [5].

There is little information concerning the fermentation of *manipueira* to obtain *tucupi* in the literature. Campos et al. [4] observed that after spontaneous fermentation periods of 12 and 24 h, their *manipueira* presented, respectively, the following characteristics: pH of 4.34 and 3.97, total acidity of 0.34 and 0.70 g lactic acid/100 mL, total HCN of 4.89 and 4.30 mg HCN/L, and free HCN of 2.90 and 2.13 mg HCN/L. Regarding the microbiological characterization performed during the preparation of the *tucupi*, *Lactobacillus plantarum* and *L. fermentum* were the two lactic acid bacteria species identified during the spontaneous fermentation of the *manipueira* [10].

Cassava roots contain the cyanogenic glycosides linamarin and lotaustralin, and these have the potential to release hydrocyanic acid (HCN) during processing, which in high concentrations may cause toxicological effects [12,13]. Biogenic amines, in turn, can be produced and accumulated during fermentation processes, mainly through the decarboxylation of free amino acids by microbial enzymes [14,15]. Generally, biogenic amines are associated with benefic neurological and vasoactive activities. However, at high concentrations, some of them may have adverse effects on human health [16,17]. For example, high histamine levels can lead to an oral burning sensation or peppery taste, hives, itching, a red rash, and hypotension, and tyramine can cause headache, sweating, agitation, chest pain, high blood pressure, and hypertensive crisis [16,17]. Another problem recently associated with histamine is histamine intolerance. This malady can lead to hypotension, respiratory dysfunction, pupil dilatation, palpitations, hypotension, and a range of allergic disorders [16,17]. Another amine associated with adverse effects is tyramine, which can cause headache, hypertension, and hypertensive crisis in individuals not taking MAOI drugs [17,18]. Biogenic amines may occur during spontaneous fermentation in *tucupi* production [10]. Furthermore, as a cassava derivative, residual amounts of HCN can be found in *tucupi,* even after cooking [11,19]. Therefore, the presence and levels of biogenic amines and HCN in *tucupi* may be related to the quality and safety of the product.

Although *tucupi* is widely used, its processing takes place mostly in a rudimentary way, without technological control and standardization, compromising the quality and stability of the product. A representative amount of *tucupi* sold in the Amazonia still comes from family farming. Thus, to understand the quality of commercial *tucupi* and contribute to the improvement of its production process, this study aimed to evaluate the quality of the *tucupi* sold in street markets and supermarkets in the city of Belém (Brazil) by determining the profile of bioactive amines and the total and free HCN contents.

## 2. Material and Methods

### 2.1. Material

Twenty-two commercial samples of *tucupi* (1 L each) were purchased in Belém, Pará, Brazil (latitude 01°27′21″ and longitude 48°30′16″). Of these, 11 samples were obtained in supermarkets (coded from 1 to 11), and these were produced using agroindustrial units registered by the Agency of Pará State Agricultural Defense (Belém, Brazil); the 11 remaining samples were obtained in street markets (coded from 12 to 22), and these were produced using non-agroindustrial units, i.e., artisanal production by family farmers. The samples were stored at −18 °C until analyses. All analyses were carried out in triplicate (*n* = 3).

With the exception of the HPLC solvents, which were LC-grade, analytical-grade reagents were used. Ultra-pure water was obtained from Milli-Q Plus (Millipore Corp., Milford, MA, USA). The organic and aqueous solvents for the HPLC analysis were filtered using HAWP and HVWP membranes, respectively (0.45 μm, Millipore Corp., Milford, MA, USA). Bioactive amine standards (spermidine trihydrochloride, agmatine sulfate, putrescine dihydrochloride, cadaverine dihydrochloride, histamine dihydrochloride, serotonin hydrochloride, tryptamine hydrochloride, tyramine hydrochloride, and 2-phenylethylamine hydrochloride) were obtained from Sigma-Aldrich Chemical Co. (St. Louis, MO, USA).

### 2.2. Physicochemical Characterization

The physicochemical characterization of the *tucupi* was carried out by determining the total acidity by titration (g lactic acid equivalent/100 mL), the pH (direct reading in a potentiometer, W12D, BEL, Monza, Italy), and the reducing and total sugars (g/100 mL) using the Lane–Eynon method [20].

### 2.3. Determination of Total and Free Hydrogen Cyanide (HCN)

Total (linamarin + acetone cyanohydrin + HCN) and free cyanide (HCN) assays were performed according to the enzymatic methodology described by Essers et al. [21] and adapted by Chisté and Cohen [19]. The total and free HCN contents were monitored via spectrophotometry at 605 nm and quantified using external eight-point analytical curves (0.5–10 µg HCN/mL) in duplicate (R^2^ ≥ 0.99). The results (*n* = 3) were expressed in mg HCN/mL *tucupi*.

### 2.4. Determination of Bioactive Amines

The determination of the free bioactive amines in the *tucupi* was carried out according to the method outlined by Brito et al. [3]. The samples were centrifuged at 8422× *g* for 20 min at 4 °C and filtered (0.45 µm pore size membrane; Millipore, Milford, MA, USA) prior to a high-performance liquid chromatography (HPLC) analysis. The amines were separated via ion-pair reverse-phase HPLC (Shimadzu, LC-10CE, Kyoto, Japan) using a Novapak C18 column (3.9 × 300 mm, 4 μm, 60 Å, Waters, Milford, MA, USA). Two mobile phases were used: 15 mM sodium octane sulfonate in 0.2 M sodium acetate, with the pH adjusted to 4.9 with glacial acetic acid (mobile phase A) and acetonitrile (mobile phase B) at a flow rate of 0.8 mL/min. The gradient elutions were 0 to 21.0 min/3–20% B, 21.0–22.0 min/20–5% B, 22.0–25.0 min/5% B, 25.0–40.0 min/5–24% B, 40.0–45.00 min/24% B, 45.0–50.0 min/24–35% B, and 50.0–51.0 min/35.3% B. Further re-equilibration was carried out at initial conditions for another 9.0 min for a total cycle time of 60.0 min until the next injection. The injection volume was 10 μL.

The amines were quantified fluorimetrically after post-column derivatization with *o*-phthalaldehyde (OPA). The post-column derivatization reagent was delivered at 0.4 mL/min. It consisted of 1.5 mL Brij-35 (Merck), 1.5 mL 2-mercaptoethanol (Sigma-Aldrich, St. Louis, MO, USA), and 0.32 g OPA (Sigma-Aldrich, St. Louis, MO, USA) in 500 mL 25 g boric acid (Merck, St. Louis, MO, USA) and 22 g KOH (Sigma-Aldrich, St. Louis, MO, USA) (pH 10.5). The column and post-column reaction apparatuses were kept at 23 ± 1 °C. The amines were identified by comparing the observed elution order, retention time, and co-elution with authentic amine standards. Quantification was accomplished via fluorimetry (340 and 445 nm excitation and emission, respectively) and interpolation in external standard curves (R^2^ ≥ 0.99) using the LCSolution software Version 1.25 SP4 (Shimadzu, Kyoto, Japan). The amine contents (*n* = 3, wet basis) were expressed in mg/L of the sample.

### 2.5. Statistical Analysis

All analyses were carried out in triplicate and the results were expressed as mean ± standard deviation (*n* = 3). The results were submitted to analysis of variance (ANOVA) and the means were compared via the Tukey test (*p* ≤ 0.05) using the Statistical Kernel Release 7.1 software.

## 3. Results and Discussion

### 3.1. Physicochemical Characteristics of the Tucupi Samples

Table 1 shows the physicochemical characteristics of the 22 *tucupi* samples analyzed in this study. According to *Normative Instruction 001/2008* of the Agency of Pará State Agricultural Defense (Brazil) [22], *tucupi* must present pH values from 3.5 to 4.3. Among the acquired *tucupi* samples, 82% had pH values below the minimum recommended limit (pH < 3.5), 55% of which were produced using agroindustrial units and 27% of which were produced using non-agroindustrial units (artisanal). Only three samples (15, 17, and 18), all from the non-agroindustrial units, presented pH values above the maximum limit recommended (pH > 4.3).

The *tucupi* samples showed pH values (2.82 to 4.67) similar to those previously reported by Chisté et al. [11] (3.0–4.35) and Campos et al. [23] (2.80–4.26), and these values allow the classification of *tucupi* as an acidic food. Only two samples (15 and 18—artisanal *tucupi*) presented pH values > 4.5. The low pH values of the *tucupi*, which might be attributed to acidification promoted by lactic acid bacteria, may be sufficient to control the growth and/or survival of pathogens [7,8]. Combined with efficient cooking, this makes *tucupi* a safe food free from biological contaminants. On the other hand, the pH values observed in the samples may favor the activity of β-glucosidase (linamarase) [24]. This enzyme is responsible for the cyanogenesis of linamarin, the cyanogenic glucoside that generates HCN in cassava and cassava-derived products, such as *tucupi* [11].

Regarding total acidity, none of the *tucupi* samples presented total acidity values lower than 0.1 g lactic acid/100 mL, the minimum content established by legislation [22], which should range from 0.1 to 0.8 g/100 mL. Only sample 8 (agroindustrial unit) and sample 20 (artisanal unit) showed total acidity higher than 0.8 g lactic acid/100 mL. The acidity values observed in the *tucupi* samples (0.15–1.36 g lactic acid/100 mL) were similar to the values reported by Chisté et al. [11] (0.3–1.0 g lactic acid/100 mL) and Campos et al. [23] (0.3 g–1.6 g lactic acid/100 mL). Cassava fermentation occurs spontaneously via the action of lactic acid bacteria and yeast, and the production of organic acids by lactic acid bacteria is superior to that of yeast [9]. For example, Penido et al. [25] verified the antagonist behavior of lactic acid bacteria and *Saccharomyces cerevisiae* and its effect on acidity and physicochemical properties during sour cassava starch production. These authors observed that although single cultures may increase acidity during fermentation, the acidity of the products obtained from single cultures did not differ from that of products obtained from the association of *Lactobacillus plantarum* and *Saccharomyces cerevisiae.*

Regarding sugar content, about 85% of the *tucupi* samples presented total sugar contents below the maximum limit recommended by the legislation [22] (1.5 g/100 mL). However, sample 11 (agroindustrial unit) and samples 17 and 21 (artisanal units) had total sugar levels above this limit and differed from the others (*p* ≤ 0.05). Low levels of total sugars can be attributed to high fermentation efficiency. In samples 1, 3, 9, and 10 (agroindustrial units) and samples 16, 20, and 22 (artisanal units), the contents of reducing sugars and total sugars did not reach the detection limit of the analytical method used. The small amount of sugars in these samples can be attributed to a long fermentation time. These results suggest a deficiency in the control of the fermentation time, not only in artisanal units but also in agroindustrial units.

On the other hand, higher levels of sugar may suggest that the cassava roots used for the *tucupi* production were the high sugar variety. It is also possible that sugar was added to these products, which is adulteration since the addition of sugar is not allowed by the legislation. Although there is no recommendation in the legislation regarding reducing sugars, we recorded reducing sugar content as data on this sugar fraction in *tucupi* are lacking in the literature. Importantly, our results indicate that the fraction of reducing sugars represents most of the sugars present in *tucupi*. Still, in samples 11, 17, and 21, which did not comply with the legislation, the reducing sugar contents were much lower than the total sugar content, so it is assumed that sucrose was added to these products.

The mean pH values, total acidity values, and reducing sugar values were similar for both the *tucupi* produced in the artisanal units and those produced in the agroindustrial units. In turn, the mean total sugar value in the *tucupi* produced in the artisanal units was approximately twice the mean observed in the *tucupi* produced in the agroindustrial units. Moreover, the *tucupi* produced in the artisanal units also showed a wider range of variation in mean total sugar value. These results suggest that control over fermentation time is less efficient in artisanal units.

Previous studies have already demonstrated a deficiency in *tucupi* standardization [11]. This can be attributed to the product processing conditions, which are very variable, especially during the fermentation and cooking stages [26]. The differences observed in the physicochemical characteristics of the studied *tucupi* samples can be attributed to agronomic and climatic factors that affect cassava cultivation, differences in cassava varieties, or the action of microbiota in the fermentation stage during *tucupi* production. Thus, our results reinforce the notion that there is a deficiency in the control of *tucupi* production and a lack of efficient supervision by the competent regulatory organs.

### 3.2. Total and Free HCN Levels in the Tucupi Samples

Cassava is considered the most important cyanogenic species in Brazil, and its cyanogenic glucoside levels range from 75 to 1000 mg HCN/kg [27,28]. Since the *manipueira* that is obtained from cassava can present high levels of HCN, it is important to determine its contents in *tucupi* to verify whether the production process eliminated it effectively.

Regarding HCN content (Table 2), 32% of the *tucupi* samples presented total cyanide contents below the minimum value observed by Chisté et al. [11] (55.58–157.17 mg HCN/kg) and Campos et al. [23] (37.67–126.21 mg HCN/L), and none of the samples showed a value higher than those observed by these authors. However, a similar profile was observed in the levels of free cyanide, which were in accordance with those observed by Chisté et al. [11] (9.47–46.86 HCN/L) and Campos et al. [23] (4.18–18.94 HCN/L).

The divergences observed in free and total HCN contents among the samples, as well as among the data presented in the literature, may be related to the natural contents of cyanogenic compounds found in cassava roots, as well as the processing conditions of *tucupi* [11,29]. The cooking time, for example, can significantly decrease the total HCN content in *tucupi* due to its high volatility under heating conditions [4]. Thus, if fermentation, which can be seen as an efficient natural detoxification process, does not take place, and an efficient cooking procedure is not carried out, the resulting *tucupi* will also exhibit high HCN contents. Campos et al. [4] observed that the longer the fermentation time, the lower the total and free HCN contents. This is probably due to the longer action of linamarase, the enzyme that hydrolyzes linamarin, which promotes HCN release, ultimately leading to HCN volatilization.

In most of the analyzed *tucupi* samples, high contents of free HCN were observed. Since HCN is volatile and *tucupi* is cooked for a long time, the high contents of free HCN can be attributed to the acidic hydrolysis of linamarin at the storage temperature of *tucupi* (≈30 °C) in the Brazilian Amazonia. Interestingly, the mean contents of total and free HCN, as well as the variation ranges, were lower in the *tucupi* produced in the non-agroindustrial units than in those produced in the agroindustrial units (Table 2). This is probably due to the longer fermentation or cooking times adopted by the farmers using the artisanal units; these longer times favor the hydrolysis and volatilization of cyanogenic compounds. In Table 2, it can also be observed that the percent ratio between free HCN and total HCN is lower in the samples produced using the artisanal units than in those produced using the agroindustrial units. This suggests that artisanal production induces a more efficient HCN elimination process.

Concerning the presence of cyanoglycosides in *tucupi*, to the best of our knowledge, no reports about poisoning after consuming *tucupi* can be found in the literature. Cereda and Vasconselos [30] explained that during the pressing of cassava roots to obtain *manipueira*, linamarin and linamarase react to generate acetone cyanohydrin and HCN. However, during the spontaneous fermentation of *manipueira*, the activity of linamarase decreases, and this immobilizes the degradation of acetone cyanohydrin in HCN. According to the same authors, as fermented *manipueira* is boiled to produce *tucupi*, the degradation of acetone cyanohydrin can be observed along with HCN volatilization and the degradation of linamarase, and this leaves only the remaining non-toxic linamarin.

However, the lethal dose (LD_50_) of HCN was reported to be 10 mg HCN/kg of body weight [31]. Therefore, based on the total HCN levels observed in the *tucupi* samples, an individual weighing 60 kg would have to consume about 6 L of *tucupi* (sample 4—the highest total HCN content) in a single meal to reach the LD_50_. However, as the presence of HCN in food may represent a risk of food poisoning, the total HCN levels we observed suggested the need to control both the fermentation and cooking times during *tucupi* production to ensure a product with safe levels. Campos et al. [4] reported low values of total and free HCN (6.97 and 1.31 mg HCN/L, respectively) after 24 h of fermentation followed by a cooking time of 40 min. These same authors also observed that the contents of total and free HCN in *tucupi* did not undergo a significant increase during 50 days of storage at 10 °C. Since amounts of free HCN were observed in all the commercial *tucupi* samples, even at low contents, our recommendation is to proceed with the cooking step before consuming *tucupi*.

### 3.3. Profiles of Free Bioactive Amines in the Tucupi Samples

This is the first study in which free bioactive amines were identified and quantified in commercial *tucupi* samples. Among the nine amines investigated, five were detected in the *tucupi*: four biogenic amines (tyramine, putrescine, histamine, and tryptamine) and one polyamine (spermidine) (Table 3). Among the biogenic amines, tyramine was detected in 18% of the samples, putrescine in all samples (100%), histamine in 72% of the samples, and tryptamine in 32% of the samples. Although *tucupi* is subjected to a cooking process after the fermentation process to eliminate HCN [4], bioactive amines are thermostable [14,32,33] and thus cannot be eliminated by heat treatment, as was observed by Brito et al. [3] during *tucupi* production under controlled laboratory conditions.

With the exception of spermidine, the mean values of the bioactive amines (tyramine, putrescine, histamine, and tryptamine) detected in the analyzed samples were higher in the *tucupi* produced in the agroindustrial units than in those produced in the non-agroindustrial units. Consequently, the mean of the sum of all the biogenic amines was five times higher in the *tucupi* produced in the agroindustrial units. This result suggests that *tucupi* produced in non-agroindustrial units is subjected to longer cooking times.

*Tucupi* is considered a favorable medium for the production of biogenic amines, with several factors contributing to their formation. There is evidence that the predominant microbiota during *manipueira* fermentation is lactic acid bacteria (LAB), which can support the low pH levels reached, especially at the end of the fermentation process [3,10,34]. The acidic media (pH 2.5–6.5) prevalent in *tucupi* (Table 1) can stimulate the accumulation of biogenic amines as a cellular defense mechanism to withstand acid stress, and it has been demonstrated that the transcription of many decarboxylase genes is induced by low pH and improves cell performances in acid conditions [17,35,36]. In fact, the activity of amino acid decarboxylases is higher in acidic conditions, i.e., at pH 4.0–5.5 [36].

Another factor that can affect the formation of biogenic amines is the fermentation temperature. It is well known that at higher temperatures, e.g., those typical of *tucupi* production (tropical area, 30–35 °C), the formation of biogenic amines increases. In addition, the food preparation method and conditions have an impact on the microflora. For example, *Enterobacteriaceae* contamination can result from inadequate processing conditions. These microorganisms have high amino acid decarboxylase activity, particularly in the production of cadaverine and putrescine, leading to their accumulation [36,37].

It is likely that by preventing microbial contamination and keeping the temperature as low as possible, the formation of biogenic amines can be minimized [36]. Furthermore, microbial modelling, with the use of a selected starter culture including microorganisms that can modulate amine formation [37] and others that can degrade (oxidize) the formed biogenic amines [35], can modulate amine formation. The use of alternative tools, e.g., pasteurization, irradiation, food additives, or preservatives (citric acid, potassium sorbate and ascorbic acid, salt) can help mitigate amine formation and build-up [36].

Although biogenic amines such as histamine and tyramine are needed for many physiological functions in humans and animals, the consumption of foods with high concentrations of these compounds can cause adverse effects. Histamine intoxication can lead to symptoms such as oral burning sensation or peppery taste, hives, itching, red rash, and anaphylactic shock. Another problem associated with histamine is histamine intolerance, which leads to hypotension, respiratory dysfunction, pupil dilatation, palpitation, and a range of allergic disorders [16,17]. According to EFSA [16], the level of histamine that may cause adverse effects to human health is 25–50 mg/meal for healthy individuals, and it is 0 mg/meal for those with histamine intolerance. Therefore, the histamine levels in sample 4 (66.85 mg/mL) could lead to histamine intoxication. However, most of the samples would be inappropriate for individuals with histamine intolerance. In addition, the presence of high levels of putrescine, typical of *tucupi*, could enhance the adverse effects of histamine [16,17,38].

When considering tyramine, levels above 600 mg/meal could cause adverse effects in healthy individuals who are not taking MAOI drugs, levels above 50 mg/meal could cause adverse effects in in individuals taking third generation MAOI drugs, and levels above 6 mg/meal could cause adverse effects in those taking classic MAOI drugs [16,17]. With these limits in mind, none of the samples analyzed in this study would cause tyramine intoxication in individuals not taking MAOI. However, individuals taking third generation MAOI drugs should be aware of the possible adverse effects [17].

The polyamine spermidine was detected in 77% of the *tucupi* samples (Table 3). The occurrence of this amine was expected since this amine is predominant in all plant-derived foods [39]. According to Dala-Paula et al. [40], spermidine (0.16–0.27 mg/100 g) and putrescine (0.08–0.61 mg/100 g) are the prevalent amines in cassava from Brazil. The presence of spermidine in food is also relevant due to the antioxidant character of polyamines [41]. Polyamines are non-toxic at concentrations normally found in food; however, they can accelerate tumor growth in patients undergoing cancer treatment, for whom a low polyamine diet is recommended [41,42,43]. According to Kalač [41], the polyamine content in a product is considered low when it is less than 10 mg/kg, high when greater than 10 mg/kg, and very high at levels above 100 mg/kg. Thus, all of the *tucupi* samples analyzed in this study presented low polyamine (spermidine) levels.

Putrescine, which is formed by the decarboxylation of the amino acid ornithine, is an obligatory intermediate in the biosynthesis of spermidine and spermine [41,44]. In fermented foods, putrescine is classified as a biogenic amine. This amine is considered rare in fresh products. However, the putrescine levels in *tucupi* could result from the activity of several groups of bacteria, mainly *Enterobacteriaceae* and *Clostridium spp*., during processing and storage under inadequate conditions [41].

It is important to highlight the fact that the samples from the agroindustrial units presented the highest total levels of biogenic amines (Table 3). Interestingly, sample 4 (Table 2), which was produced in an agroindustrial unit, showed the highest total HCN (114.66 mg/L) and free HCN (38.38 mg HCN/L) contents, as well as the highest biogenic amine content (78.22 mg/L). These results indicate that the fermentation and cooking processes used to produce this *tucupi* sample were deficient.

The present results point to high total and free HCN levels, as well as high biogenic amine concentrations, in most of the *tucupi* samples we evaluated. Thus, effective control in the fermentation and cooking stages during *tucupi* production is of paramount importance to ensure low total and free HCN levels in the product, as has already been reported by Campos et al. [4]. Regarding biogenic amines, since they are thermostable, good manufacturing and manipulation practices (GMMP) in the different *tucupi* processing stages and effective control during fermentation are the most important controls to ensure low levels of these amines in *tucupi*.

## 4. Conclusions

In this study, we evaluated the physicochemical characteristics, HCN levels, and—for the first time—the profiles and contents of bioactive amines in commercially available *tucupi* samples obtained in Northern Brazil and produced using both agroindustrial and non-agroindustrial units. Regarding the physicochemical properties, differences were observed between the samples due to the very different processing conditions of the products. Four biogenic amines were detected (tyramine, putrescine, histamine, and tryptamine), and their presence may be indicative of the poor quality of the raw material used and/or poor manufacturing practices during the production of the *tucupi*. The samples produced using the agroindustrial units presented the highest concentrations of total and free HCN and of biogenic amines, indicating the inefficiency of the inspections carried out by the control agencies as well as deficiencies in the control of the fermentation and cooking stages and of the manipulation practices during *tucupi* production. The great variability observed in the properties of the commercial *tucupi* samples highlighted the need to standardize the conditions for processing and to carry out better controls during *tucupi* production. The data hitherto obtained are relevant for practical purposes; however, more in-depth studies are necessary to better understand the transformations that occur during the fermentation stage. Consequently, it will be possible to define optimal conditions for the production of *tucupi* which will ensure the maintenance of the product’s characteristics as well as the elimination or minimum levels of potentially toxic compounds (cyanide and amines).

## Figures and Tables

**Table 1:** Physicochemical characterization of *tucupi* available for consumption in Northern Brazil (Belém, Pará State).

Source/Sample	pH	Total Acidity (g Lactic Acid/100 mL)	Total Sugars (g/100 mL)	Reducing Sugars (g/100 mL)
Agroindustry				
1	3.39 ± 0.01 ^mn^	0.16 ± <0.01 ^mn^	nd	nd
2	3.40 ± 0.01 ^m^	0.19 ± <0.01 ^ljm^	0.44 ± 0.01 ^gh^	nd
3	3.27 ± 0.02 ^o^	0.40 ± <0.01 ^g^	nd	nd
4	3.66 ± 0.01 ^i^	0.56 ± 0.01 ^e^	0.50 ± 0.02 ^gh^	0.50 ± 0.01 ^def^
5	4.30 ± 0.01 ^d^	0.18 ± <0.01 ^lmn^	0.66 ± 0.02 ^fg^	0.64 ± 0.01 ^bcde^
6	3.26 ± 0.01 ^o^	0.24 ± <0.01 ^hi^	0.32 ± 0.01 ^h^	0.22 ± 0.01 ^f^
7	3.06 ± 0.02 ^p^	0.67 ± 0.01 ^c^	0.79 ± 0.01 ^ef^	nd
8	3.52 ± 0.01 ^j^	1.07 ± 0.02 ^b^	0.28 ± 0.01 ^h^	0.23 ± 0.01 ^f^
9	2.82 ± 0.04 ^q^	0.37 ± 0.01 ^g^	nd	nd
10	4.04 ± 0.01 ^f^	0.59 ± 0.01 ^de^	nd	nd
11	4.22 ± 0.02 ^e^	0.38 ± 0.02 ^g^	2.71 ± 0.14 ^b^	2.33 ± 0.47 ^a^
Mean	3.54 ± 0.47	0.44 ± 0.27	0.81 ± 0.86	0.78 ± 0.88
Range	2.82–4.30	0.16–1.07	0.00–2.71	0.00–2.33
Artisanal				
12	3.93 ± 0.01 ^g^	0.17 ± 0.01 ^mn^	0.92 ± 0.03 ^de^	0.69 ± 0.22 ^bcd^
13	3.47 ± 0.01 ^l^	0.14 ± <0.01 ^n^	1.04 ± 0.04 ^d^	1.02 ± 0.05 ^b^
14	3.85 ± 0.01 ^h^	0.22 ± <0.01 ^ijl^	0.60 ± 0.02 ^fg^	0.57 ± 0.02 ^cdef^
15	4.67 ± 0.01 ^a^	0.24 ± <0.01 ^hi^	0.73 ± 0.02 ^ef^	0.60 ± 0.06 ^cdef^
16	3.45 ± 0.01 ^l^	0.48 ± 0.01 ^f^	nd	nd
17	4.51 ± 0.01 ^c^	0.23 ± <0.01 ^ij^	4.35 ± 0.29 ^a^	0.27 ± 0.01 ^ef^
18	4.59 ± 0.02 ^b^	0.27 ± <0.01 ^h^	0.94 ± 0.07 ^de^	0.91 ± 0.06 ^bc^
19	3.47 ± 0.01 ^l^	0.15 ± 0.01 ^n^	1.14 ± 0.06 ^d^	1.04 ± 0.10 ^b^
20	3.02 ± 0.02 ^p^	1.36 ± 0.03 ^a^	nd	nd
21	3.39 ± 0.02 ^m^	0.60 ± 0.03 ^d^	2.07 ± 0.06 ^c^	0.56 ± 0.02 ^cdef^
22	3.35 ± 0.02 ^n^	0.40 ± 0.03 ^g^	nd	nd
Mean	3.79 ± 0.57	0.39 ± 0.35	1.47 ± 1.24	0.71 ± 0.27
Range	3.02–4.67	0.14–1.36	0.00–4.35	0.00–1.04

Values (mean ± standard deviation, *n* = 3, fresh weight, representing two batches for each sample) with equal letters in the same column do not differ statistically from each other (Tukey’s test at 5% significance). nd: not detected.

**Table 2:** Total and free HCN levels in the *tucupi* samples.

Source/Sample	Cyanide (mg HCN/L)		% Free/Total
	Total	Free	
Agroindustry			
1	60.06 ± 0.31 ^d^	20.52 ± 0.06 ^d^	34.2
2	49.51 ± 0.38 ^e^	1.16 ± 0.01 ^o^	2.3
3	79.22 ± 2.45 ^c^	30.92 ± 0.05 ^c^	39.0
4	114.66 ± 3.29 ^a^	38.38 ± 0.61 ^a^	33.5
5	107.09 ± 4.06 ^ab^	34.27 ± 1.22 ^b^	32.0
6	32.03 ± 0.61 ^hij^	8.27 ± 0.14 ^jl^	25.8
7	43.13 ± 1.61 ^efg^	18.54 ± 0.20 ^e^	43.0
8	67.05 ± 1.15 ^d^	16.77 ± 0.20 ^f^	25.0
9	8.87 ± 0.77 ^l^	3.33 ± 0.01 ^n^	37.5
10	40.85 ± 2.83 ^efgh^	3.91 ± 0.09 ^n^	9.6
11	81.55 ± 0.69 ^c^	14.65 ± 0.20 ^g^	18.0
Mean	62.18 ± 31.93	17.34 ± 12.86	27.26 ± 12.76
Range	8.87–114.66	3.33–38.38	2.3–43.0
Artisanal			
12	103.19 ± 6.66 ^b^	17.97 ± 0.61 ^ef^	17.4
13	37.18 ± 2.53 ^fghi^	8.17 ± 0.33 ^l^	21.97
14	59.79 ± 3.29 ^d^	12.01 ± 0.51 ^h^	20.1
15	23.81 ± 1.34 ^j^	0.80 ± 0.06 ^o^	3.4
16	47.40 ± 1.22 ^e^	8.00 ± 0.04 ^lm^	16.9
17	64.45 ± 1.45 ^d^	10.04 ± 0.32 ^i^	15.6
18	12.72 ± 0.23 ^l^	0.84 ± 0.09 ^o^	6.6
19	27.87 ± 0.23 ^ij^	6.59 ± 0.11 ^m^	23.6
20	42.05 ± 1.45 ^efg^	9.75 ± 0.11 ^ij^	23.2
21	35.44 ± 2.83 ^ghi^	8.32 ± 0.14 ^jl^	23.5
22	46.21 ± 2.60 ^ef^	17.32 ± 0.20 ^ef^	37.5
Mean	45.46 ± 24.36	9.07 ± 5.50	19.07 ± 9.09
Range	12.72–103.19	0.84–17.97	3.4–37.5

Values (mean ± standard deviation, *n* = 3, fresh weight, representing two batches for each sample) with equal letters in the same column do not differ statistically from each other (Tukey’s test at 5% significance).

**Table 3:** Profiles and levels of bioactive amines in the *tucupi* samples.

Source/Sample	Bioactive Amines (mg/L)					
	Tyramine	Putrescine	Histamine	Tryptamine	Spermidine	Total *
Agroindustry						
1	nd	31.79 ± 1.28 ^a^	1.55 ± 0.11 ^cd^	nd	0.44 ± 0.15 ^g^	33.34 ± 1.39
2	nd	6.24 ± 0.29 ^e^	1.56 ± 1.03 ^cd^	nd	nd	7.80 ± 1.32
3	4.21 ± 0.04 ^a^	7.01 ± 0.22 ^e^	1.00 ± 0.04 ^cd^	nd	0.80 ± 0.12 ^efg^	12.22 ± 0.30
4	0.83 ± 0.02 ^c^	10.54 ± 0.49 ^d^	66.85 ± 2.78 ^a^	nd	1.64 ± 0.06 ^bcd^	78.22 ± 3.29
5	nd	2.29 ± 0.05 ^hijl^	nd	2.94 ± 0.19 ^a^	0.92 ± 0.01 ^efg^	5.23 ± 0.24
6	nd	16.11 ± 0.34 ^c^	4.01 ± 0.19 ^c^	2.53 ± 0.14 ^ab^	0.67 ± 0.01 ^fg^	22.65 ± 0.67
7	3.66 ± 0.16 ^b^	2.49 ± 0.13 ^hijl^	13.62 ± 0.09 ^b^	0.81 ± 0.03 ^c^	nd	20.58 ± 0.41
8	nd	1.60 ± 0.08 ^ijl^	16.79 ± 1.59 ^b^	nd	nd	18.39 ± 1.67
9	nd	23.07 ± 0.87 ^b^	1.95 ± 0.16 ^cd^	1.77 ± 0.12 ^d^	nd	26.79 ± 1.15
10	nd	2.86 ± 0.14 ^ghij^	15.98 ± 0.53 ^b^	nd	1.72 ± 0.35 ^bc^	18.84 ± 0.67
11	nd	2.77 ± 0.04 ^hijl^	nd	1.65 ± 0.05 ^d^	1.03 ± 0.02 ^efg^	4.42 ± 0.09
Mean	2.90 ± 1.81	9.71 ± 9.98	13.70 ± 21.00	1.94 ± 0.83	1.03 ± 0.48	22.59 ± 20.55
Range	0.83–4.21	1.60–31.79	1.00–66.85	0.81–2.94	0.44–1.72	5.23–78.22
Artisanal						
12	nd	1.77 ± 1.03 ^ijl^	nd	nd	2.20 ± 0.14 ^ab^	1.77 ± 1.03
13	nd	2.83 ± 0.07 ^ghij^	nd	nd	1.39 ± 0.16 ^cde^	2.83 ± 0.07
14	nd	1.23 ± 0.26 ^jl^	0.49 ± 0.09 ^d^	nd	1.08 ± 0.06 ^def^	1.72 ± 0.35
15	nd	3.22 ± 0.05 ^ghi^	1.14 ± 0.03 ^cd^	nd	1.68 ± 0.13 ^bcd^	4.36 ± 0.08
16	nd	1.00 ± 0.02 ^l^	2.21 ± 0.09 ^cd^	nd	nd	3.21 ± 0.11
17	nd	1.25 ± 0.03 ^jl^	1.64 ± 0.19 ^cd^	nd	2.48 ± 0.25 ^a^	2.89 ± 0.22
18	nd	5.29 ± 0.53 ^ef^	0.81 ± 0.06 ^cd^	nd	0.80 ± 0.11 ^efg^	6.10 ± 0.59
19	nd	4.65 ± 0.22 ^fg^	1.13 ± 0.51 ^cd^	nd	0.85 ± 0.18 ^efg^	5.78 ± 0.73
20	nd	5.21 ± 0.12 ^ef^	nd	0.62 ± 0.15 ^c^	0.66 ± 0.04 ^fg^	5.83 ± 0.27
21	0.49 ± 0.08 ^c^	3.94 ± 0.24 ^fgh^	1.51 ± 0.21 ^cd^	nd	0.56 ± 0.13 ^fg^	5.94 ± 0.53
22	nd	1.78 ± 0.03 ^ijl^	nd	2.10 ± 0.02 ^bd^	0.42 ± 0.01 ^g^	3.88 ± 0.05
Mean	0.49 ± 0.00	2.92 ± 1.64	1.28 ± 0.57	1.36 ± 1.05	1.21 ± 0.71	4.03 ± 1.68
Range	0.49	1.00–5.29	0.49–2.21	0.62–2.10	0.42–2.48	1.72–6.10

Values (mean ± standard deviation, *n* = 3, fresh weight, representing two batches for each sample) with equal letters in the same column do not differ statistically from each other (Tukey’s test at 5% significance). nd = not detected. * Total = sum of the biogenic amines (tyramine + putrescine + histamine + tryptamine).

## Data Availability

Data are contained within the article.
